# Performance Investigation of Micromixer with Spiral Pattern on the Cylindrical Chamber Side Wall

**DOI:** 10.3390/mi14071303

**Published:** 2023-06-25

**Authors:** Shuang Yang, He Zhang, Shuihua Yang, Yunlong Zheng, Jianan Wang, Rongyan Chuai

**Affiliations:** 1 School of Information Science and Engineering, Shenyang University of Technology, Shenyang 110870, China; yangshuang@smail.sut.edu.cn (S.Y.); shielder11345@smail.sut.edu.cn (J.W.); chuairongyan@sut.edu.cn (R.C.); 2 AVIC General Technology Co., Ltd., Beijing 100095, China; yangshuihua@cavige.com; 3 Shenyang AVIC General Technology Co., Ltd., Shenyang 110034, China; zhengyunlong@cavige.com

**Keywords:** spiral pattern, cylindrical mixing chamber, asymmetric inlet, mixing efficiency

## Abstract

In this paper, a sequence of passive micromixers with spiral patterns on the side wall of cylindrical chambers are designed, optimized, prepared and tested. The simulation studies show that the vortex magnitude and continuity in the mixing chamber are the most important factors to determine mixing performance, while the inlet position and structural parameters are secondary influences on their performance. According to the above principles, the performance of a micromixer with a continuous sidewall spiral finally wins out. The total mixing length is only 14 mm, but when Re = 5, the mixing index can reach 99.81%. The multi-view visual tests of these mixer chips prepared by 3D printing are consistent with the simulation results. This paper provides a new idea for optimizing the micromixer with spiral patterns on the side wall and the problems of floor area and pressure loss are significantly improved compared to the conventional spiral structure.

## 1. Introduction

Micro total analysis system (μTAS) has become a powerful tool for micro-scale research in medicine, biology, chemistry and other fields [1]. As an important part of μTAS, the performance improvement of the micromixer has also received extensive attention [2]. Micromixers can be classified into passive and active mixers. For active mixers, an external force, such as magnetic force [3], acoustic force [4], electrophoresis [5] or Lorentz force [6], is applied to the fluid for mixing. Active micromixers can achieve excellent mixing efficiency, but these additional drive components, such as electrodes [7] and rotors [8], pose a major obstacle for integrated applications. In contrast, passive micromixers, which are very suitable for on-chip integration, can achieve mixing under laminar flow conditions with extremely low flow velocity only depending on the delicate structure of the microchannels. At present, many successful design principles have already been developed for passive micromixers [9], such as adding obstacles periodically in the microchannel to increase the fluid disturbance [10]; splitting the fluid and then recombining them again to increase the diffusion areas [11]; Tesla mixing structures that can generate lateral or even reverse tributaries [12]; and chaotic flows which can be obtained by squeezing and stretching operations on fluid [13,14].

In addition, a spiral structure capable of generating a Dean vortex is one of the most effective passive micromixer design philosophies [15,16]. In 2017, Duryodhan et al. [17] investigated the cross-sectional aspect ratio effect of planar spiral microchannels on mixing characteristics experimentally and numerically. The results show that spiral microchannels with higher aspect ratios had better mixing efficiencies compared to those with smaller aspect ratios. In the same year, Lakshmi et al. [18] found that the cross-sectional geometries of planar spiral channels, especially in the case of irregular shapes, such as semi-circular and trapezoidal profiles, are an important factor in tuning the Dean vortex strength and dictate the mixing performance. In 2018, Pouya et al. [19] proposed a spiral micromixer with expansion and contraction components. Comparisons to the spiral mixers without expansion components showed that this mixer provides up to 92% of homogeneity at Re 1.0 and its performance was significantly improved. However, the area of this structure exceeds 70 mm^2^. In 2021, Binfeng Yin et al. [20] studied the fluid flow characteristics of the Archimedes spiral, Fermat spiral, and hyperbolic spiral structures with various channel widths and the Reynolds number (Re) ranging from 0 to 10 via numerical simulation and visualization experiments. In 2022, Bahrami et al. [21] designed a spiral micromixer with sinusoidal channel walls. Compared to a simple spiral micromixer, the degree of mixing can increase by 99.11% when Re = 50. Although the sinusoidal channel walls structure can greatly improve the performance of a spiral mixer, the pressure drop problem that is related to the amplitude of sinusoidal walls become an obstacle to on-chip integration.

With the advancement of MEMS processing technology, the successful preparation of 3D spiral structures [22] provided further improvements in the performance of the mixer. In 2016, Beugelaar et al. [23] developed a 3D circular helical micromixer. They investigated the influence of double helix and triple helix structure parameters on mixer performance, and the results showed that when 1 < Re < 10, the Dean number in this low range was still capable of convective mixing. In 2021 Farahinia et al. [24] investigated the effect of cross-section shape and input angles on the performance of micromixers at low Re numbers by numerical simulation. The results showed that the mixing index of circular cross-sections was superior to other cross-sections. In 2022 Wang et al. [25] presented a 3D spiral micromixer fabricated by 3D printing technology. Compared with the planar structure, the 3D spiral structure can increase the mixing efficiency effectively. However, the mixing efficiency is in relation to the diameter and number of turns of the spiral structure; when the mixing efficiency achieves 0.91, the spiral diameter has already reached 5 mm. It is evident that scholars have found ways to improve the Dean vortex in the planar or 3D spiral structure, and enhanced the micromixer performance successfully through an in-depth study of parameters such as channel depth width ratio, cross-section shape, and diameter of the spiral structure. Unfortunately, the problems such as large floor areas and huge pressure drops have not been solved.

In this paper, inspired by the phenomenal design of Stroock in 2002 [26], which later became known as the staggered herringbone mixer (SHM) [27,28,29,30,31,32], several passive micromixers with spiral patterns on the side wall of cylindrical chambers are designed. Then, the relationships between the side wall spiral pattern and shear rate, vorticity magnitude and mixing index are analyzed in-depth and summarized based on energy gradient theory. Finally, with the help of visual testing, the correctness of the above design rules is verified.

## 2. Mixer Design and Simulation Model Establishment

### 2.1. Micromixer Design

**Figure 1:**
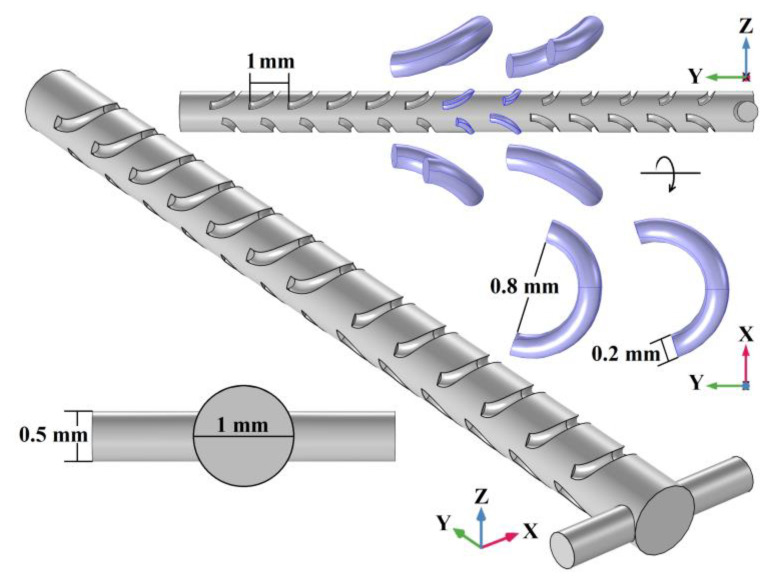
The micromixer with spiral staggered herringbone (SSH) patterns on the side wall.

According to the 3D SHM optimization structure design for a rectangular mixing chamber [33,34], a cylindrical micromixer structure with side wall spiral patterns that are similar to a staggered herringbone is presented in Figure 1. The T-type inlet is composed of two symmetrically placed cylinders with a diameter of 0.5 mm. The cylindrical mixing chamber is 14 mm in length and 1 mm in diameter. The spiral staggered herringbone (SSH) patterns are composed of a left-handed spiral and a right-handed spiral, which have a major diameter of 1.2 mm, a minor diameter of 0.2 mm and an axial pitch of 1 mm. The first six structures of the SSH array at the top of the mixing chamber consist of 0.3 turns in the left-handed spiral and 0.2 turns in the right-handed spiral, while the rest of the structures are turned over, with the left-handed spiral changed to 0.2 turns and the right-handed spiral changed to 0.3 turns. The SSH structures at the bottom of the mixing chamber are complementary to the top, when 0.3 turns in the left-handed spiral at the top there are 0.2 turns in the right-handed spiral at the bottom, while 0.2 turns in the right-handed spiral at the top there is 0.3 turns in the left-handed spiral at the bottom.

### 2.2. Establishment of the Simulation Model

In order to characterize the performance of micromixers with SSH structures, a numerical simulation model is established with COMSOL Multiphysics 6.0. The basic parameters of the simulation model are shown in Table 1.

According to the basic model parameters in Table 1 and the SSH micromixer structure in Figure 1, the fluid motion state in the mixer determined by the Reynolds number (Re) is calculated by Equation (1) first. At the T-type inlet, the structural characteristic scale (L_inlet_) is 0.5 × 10^−3^ m, Re = 2.5 ≪ 2300. In the cylindrical mixing chamber, the structural characteristic scale (L_chamber_) is 1 × 10^−3^ m, Re = 5 ≪ 2300.

(1) Re =ρuLη

The calculation of the Re proves that the fluid movement inside the mixer is a typical laminar flow, so the fluid flow of the model can be described by the Navier–Stokes equations as shown in Equation (2). Here, *ρ* is the density (kg/m^3^), *u* is the velocity (m/s), *μ* is the viscosity (N·s/m^2^) and *p* is the pressure (Pa). The modeled fluid is water with a viscosity of 1 × 10^−3^ N·s/m^2^ and a density of 1000 kg/m^3^.

(2) $$\rho u\cdot \nabla u=-\nabla p+\nabla \mu \left(\nabla u+{\left(\nabla u\right)}^{T}\right)\;\nabla u=0$$

The mass transport of the model is described by the convection–diffusion Equation (3). Here, *D* is the diffusion coefficient (m^2^/s); *c* is the concentration of the components (mol/m^3^); and *R* is the reaction rate between components. *R* = 0, when no reactions between the mixing fluids, and the mass transport between fluids is determined by both convection diffusion (u∇c) and molecular diffusion ($D\nabla^{2}c$).

(3) $$D\nabla^{2}c-u\nabla c+R=0$$

**Figure 2:**
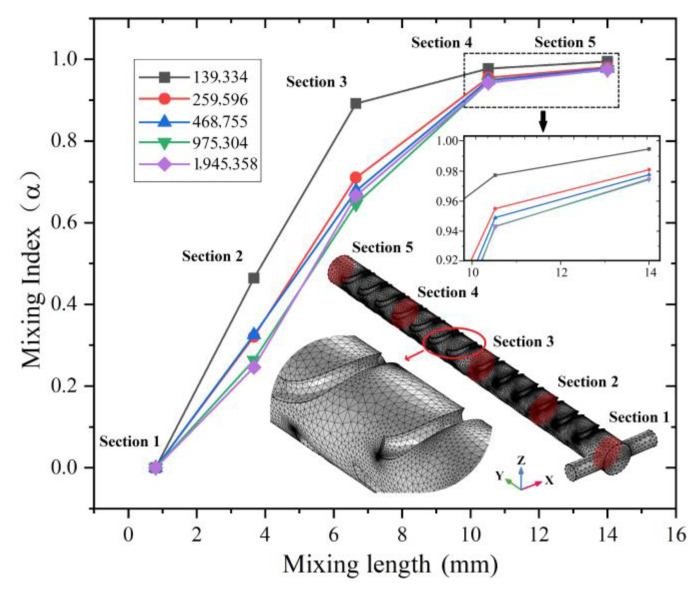
Mesh independence analysis for the mixing indexes of SSH mixer and the mesh used in the current model.

After determining the equations, the uniform velocities are employed at the T-type inlet. No-slip boundary conditions are assumed at the inner walls, and the zero pressure boundary condition is employed at the outlet, while ordered and clear unstructured tetrahedral elements are selected as mesh elements. Then, to ensure the simulation accuracy and save computing resources, the independence of the mesh is characterized by calculating the mixing index of the SSH mixer. As shown in Figure 2, in order to reflect the mixing index accurately, the cylindrical mixing chamber is divided into 4 equal parts along the *Y* axis, and then the mixing indexes of 5 sampling cross sections including the mixer outlet are calculated according to Equation (4). The coordinates of the sampling section along the *Y* axis are 0.8 mm (section 1, no SSH structures), 3.67 mm (section 2, after 3 SSH structures), 6.65 mm (section 3, after 6 SSH structures), 10.52 mm (section 4, after 10 SSH structures) and 14 mm (section 5, after 13 SSH structures).

(4) $$\alpha =1-\sqrt{\frac{\sigma^{2}}{\sigma_{\max}^{2}}}$$

Here, *σ*_max_ is the concentration variance that the fluid is not mixed at the micromixer inlet (section 1 in Figure 2), *σ* is the variance in the concentration, which can be defined by Equation (5). In Equation (5), *C_i_* is the concentration of the statistical area; *N* is the number of samples in the statistical area; and $\bar{C}$ = 1.5 mol/L is the average of statistics.

(5) $$\sigma =\sqrt{\frac{1}{N}\sum_{i=1}^{N}{\left(C_{i}-\bar{C}\right)}^{2}}$$

As shown in Figure 2, when *u* = 5 × 10^−3^ m/s, five mesh solutions with different numbers of nodes ranging from about 139,334 to 1,945,358 are tested for mesh independence. Due to further refinement of the mesh produced less than 1% change in the mixing index, when the maximum element growth rate is 1.15 and the curvature factor is 0.6, the mesh system with 975,304 nodes is suitable for the current model scale. Under current conditions, the mixing index of the SSH mixer can reach 97.42%.

## 3. Results and Discussions

### 3.1. Performance Analysis and Structural Optimization

**Figure 3:**
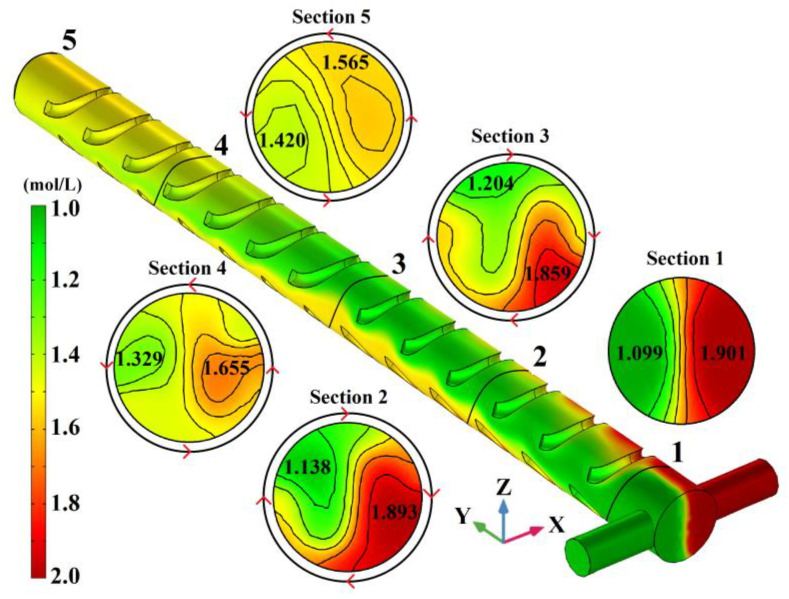
The SSH mixer concentration distribution of mixing chamber surface and inner section.

By utilizing the above model, when *u* = 5 × 10^−3^ m/s, the SSH mixer concentration distribution of the mixing chamber surface and inner sections are obtained and shown in Figure 3. The different concentrations of fluid to be mixed are distinguished by the color green (1 mol/L) and red (2 mol/L), and the color legend between them indicates the mixing degree. The black closed curve in the figure is the isoconcentration curve. As shown in Figure 3, when the green and red liquids just enter the mixing chamber, the concentration distribution on the surface of the chamber and section 1 can be regarded as an extension of the symmetrical T-shaped inlet. The interface between the two colors is distinct and located in the Y-Z plane of the mixing chamber’s central axis. After undergoing 3 groups SSH structures composed of 0.3 turns in the left-handed spiral and 0.2 turns in the right-handed spiral at the mixing chamber top while 0.2 turns in the right spiral and 0.3 turns in the left spiral at the bottom, the concentration distribution on the surface of the chamber and section 2 show that the liquid is rotating clockwise. The green liquid moves from the X-negative to Z-positive, while the red liquid moves from X-positive to Z-negative. The rotation makes the liquid interface twist and stretch, which accelerates mass transfer and diffusion. And the reduction of cross-sectional concentration difference is from 0.802 mol/L (section 1) to 0.755 mol/L (section 2). In section 3, the green liquid has occupied the top of the mixing chamber (Z-positive), while the red liquid is located at the bottom of the mixing chamber (Z-negative). Obviously, the clockwise rotation continuously enhanced after experiencing another 3 SSH structures with the same distribution again. So, the twisting and stretching of the two-color interface is also intensified, and the concentration difference is reduced to 0.655 mol/L (section 3). After section 3, the distribution of the SSH structures changed, the left-handed spiral decreased to 0.2 turns and the right-handed spiral reached 0.3 turns at the mixing chamber top, while the SSH structures at the bottom of the chamber changed in the opposite direction. By passing 4 complementary SSH structures, the rotation of the two-color liquid seemed to stop in place, and the green liquid did not continue to move toward the X-positive; meanwhile, the red liquid did not move to the X-negative also. The clockwise rotation law is broken. The concentration distribution of section 4 is similar to section 1. The two-color liquid in the Y-Z plane restored the symmetrical distribution on both sides of the mixing chamber’s central axis. The concentration difference is reduced to 0.326 mol/L. In section 5, after passing through another 4 complementary SSH structures, the orange liquid that evolved from the red liquid rotated towards Z-positive, while the yellow-green liquid that evolved from the green liquid rotated towards Z-negative. The trend of the counterclockwise rotation of liquid is obvious. The section concentration difference is further reduced to 0.145 mol/L. In short, when the liquid meets the SSH structure, the liquid in the mixing chamber rotates, and the rotation direction is related to the spiral distribution of the SSH structures.

**Figure 4:**
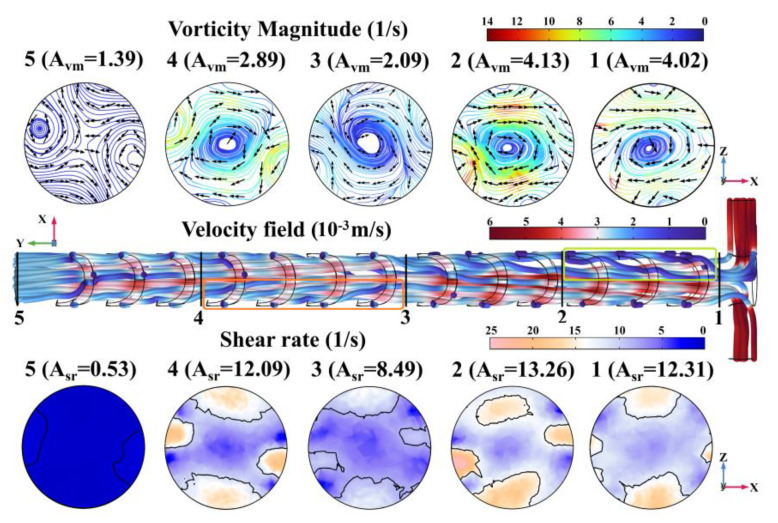
The SSH mixer concentration distribution of mixing chamber surface and inner section.

In order to clarify the relationship between the sidewall spiral distribution and fluid rotating in the mixing chamber, the vorticity magnitude (including the arrow vortex line and vorticity streamline) and shear rate in the sampling cross-sections are given in Figure 4, along with the velocity field in the top view of the mixing chamber. On the vortex line, the direction of the tangent line at any point is the same as the directions of the flow vorticity at that particle element, while the vorticity streamlines are indicated by a rainbow color table, with dark red indicating the highest and dark blue indicating the lowest. The fluid velocity field is the vector velocity distribution at the front of fluid flow. The velocity field in Figure 4 is indicated by the wave color table, with dark red indicating the highest and dark blue indicating the lowest. The shear rate is the velocity gradient caused by the different velocities in the laminar layer. The shear rate in Figure 4 is indicated by the twilight color table, with twilight indicating the highest and dark blue indicating the lowest. The vorticity and shear rate at every node in the sampling cross-sections can be directly exported from COMSOL Multiphysics 6.0, so the average vorticity magnitude (A_vm_) and the average shear rate (A_sr_) of each sampling cross-section are calculated and marked in the figure to accurately characterize the relationship between them.

In section 1, the arrow vortex lines indicate that there are already clockwise vortexes around (A_vm1 (SSH)_ = 4.02 (1/s)) the central axis of the mixing chamber in the Y direction when the liquid does not pass through the SSH structures. Meanwhile, the velocity field changes from dark red to light blue immediately after entering the mixing chamber from the T-type inlet, which indicates a rapid decrease in velocity near section 1. There are two main reasons for the velocity decrease: one is the sudden widening of the mixer structure and the other is the two-color liquid impinging in the X-direction near the symmetrical T-type inlet [35]. The shear rate distribution shows that the sudden decrease in velocity has formed a velocity gradient (A_sr1 (SSH)_ = 12.31 (1/s)) in section 1. The subsequent fluid will detour around the low-velocity fluid because of the continuity of the fluid medium, and then the vortex is generated. According to the energy gradient theory in the literature [36], if the mixing chamber sidewall is smooth and cannot provide enough mechanical energy gradients in the normal direction of the streamline to amplify the initial vortex, the larger loss of mechanical energy in the streamline direction will absorb the initial disturbance and restore the original laminar flow state. In section 2, after three groups of SSH sidewall structures, the increasing vorticity magnitude (A_vm2 (SSH)_ = 4.13 (1/s)) indicates that the initial clockwise vortexes are amplified. The velocity field shows that, due to the flow resistance caused by the SSH sidewall structures, especially for a left-handed spiral with large turns, the near-wall fluid velocity is slow (the dark blue streamlines that indicate the slower-flowing are marked by the light green rectangle in Figure 4), while the slower-flowing layer would become a dragger of the faster-flowing layer near the center of the mixing chamber. So, the velocity gradient (A_sr2 (SSH)_ = 13.26 (1/s)) between the near-wall fluid and the paraxial fluid is formed, amplifying the laminar vortexes [36]. In section 3, after six groups of SSH sidewall structures, the decrease in vorticity magnitude (A_vm3 (SSH)_ = 2.09 (1/s)) signifies that the vortices are becoming frail. In the vicinity of section 3, the velocity field along the *Y*-axis turns light blue. The change in liquid velocity is due to the reversal of the SSH structure in the second half of the mixing chamber. The original left-handed spirals with large turns are changed into the right-handed spirals with small turns, the sidewall resistance to flow reduces, and the fluid accelerates forward along the *Y*-axis. While the original right-handed spirals with small turns become the left-handed spirals with large turns, the sidewall resistance to flow increases and the fluid decelerates forward along the *Y*-axis. The acceleration and the deceleration of the liquid occur at the same time in the different positions of the mixing chamber, which makes the velocity gradient tend to be consistent and then the shear rate (A_sr3 (SSH)_ = 8.49 (1/s)) decreases also. In section 4, in passing 4 complementary SSH structures, the vorticity magnitude (A_vm4 (SSH)_ = 2.89 (1/s)) increases again, which shows that the vortexes are amplified once more. The arrow vortex line shows that the vortexes become counterclockwise with the overturning of the SSH structure. The velocity field shows that, after passing through the large turns left-handed spiral with high flow resistance in the complementary SSH structure, the liquid streamlines near the sidewall change from light blue to blue, which indicates that the flow velocity is further reduced (marked with an orange rectangular box in Figure 4). While passing the right-handed spiral structure with relatively low flow resistance, the liquid streamlines change from light blue to grayish red, which indicates that the flow velocity is further increased. With the increase in velocity difference, the shear rate in section 4 also increases (A_sr4 (SSH)_ = 12.09 (1/s)), which is the reason for the re-amplification of the vortexes. In section 5, at the outlet of the mixing chamber, the vorticity magnitude is the lowest (A_vm5 (SSH)_ = 1.39 (1/s)) among the five sampling sections, which indicates that the vortexes are about to disappear. The velocity field shows that, when passing through the mixing chamber with short smooth sidewalls, the color of all the liquid streamlines turns gray-blue, indicating that the velocity field tends to be consistent again. Therefore, the shear rate also drops rapidly (A_sr5 (SSH)_ = 0.53 (1/s)), which cannot be used as a vortexes amplifier.

**Figure 5:**
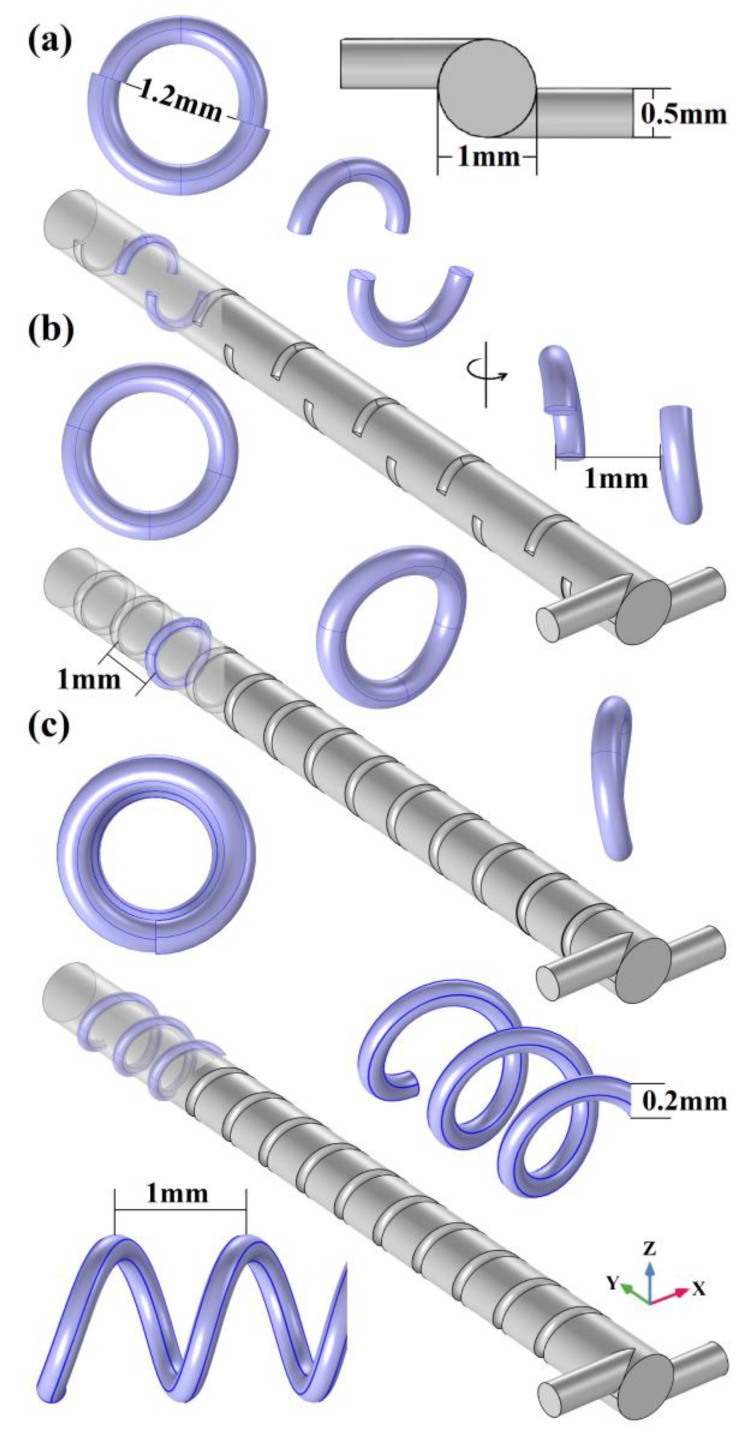
The mixer structures with different spiral pattern on the mixing chamber sidewall. (**a**) Top and bottom staggered spiral mixer; (**b**) closed staggered spiral mixer; (**c**) continuous spiral mixer.

In order to conduct an in-depth analysis and generalize the rules of the sidewalls spiral patterns influence on the mixing effect, the top and bottom staggered spiral mixer (TBSS), the closed staggered spiral mixer (CSS) as well as the continuous spiral mixer (CS), are designed. As shown in Figure 5a, TBSS comprises spiral patterns with different rotation directions, which are placed in a staggered arrangement at the top and bottom of the mixing chamber. As shown in Figure 5b, CSS means that the spiral patterns with different rotation directions are combined into a new circumference and placed on the sidewall of the mixing chamber. As shown in Figure 5c, CS means that 13-turn continuous left-handle spirals are placed on the mixing chamber sidewall. To create a bigger initial vortex, a horizontally asymmetric T-type inlet with one side at the upper left as well as the other side at the lower right is chosen to replace the original symmetrical inlet of SSH [37]. In addition to adjusting the inlet position, the structure parameters of the above 3 mixers, including cylindrical inlets (0.5 mm in diameter), cylindrical mixing chambers (14 mm in length and 1 mm in diameter), the number of spiral patterns (13), as well as the minor diameters (0.2 mm) and major diameters (1.2 mm) of spiral patterns, are consistent with SSH mixer.

**Figure 6:**
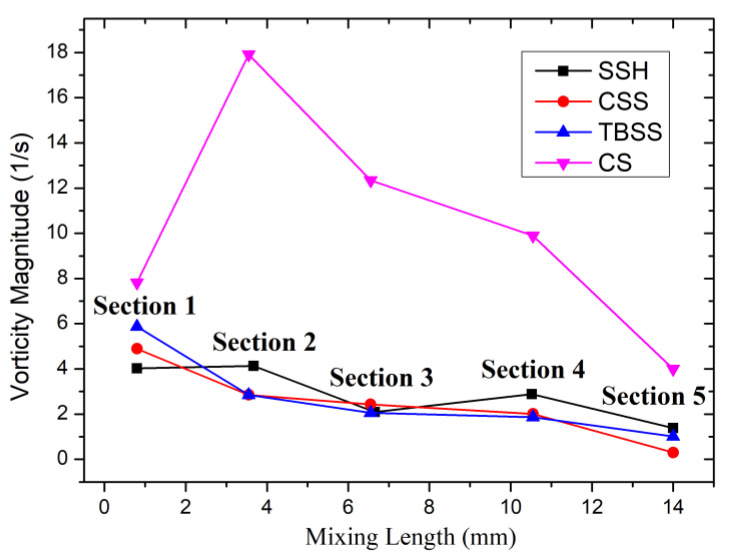
The vorticity magnitude curves of micromixer with different spiral patterns.

The vorticity magnitude curves of the micromixer with different spiral patterns are shown in Figure 6. The positions of the sampling sections in the curve are the same as in Figure 2. As shown in section 1 of the curve, compared with the symmetrical T-type inlet of the SSH structure (A_vm1 (SSH)_ = 4.02 (1/s)), all the 3 vorticity magnitudes of the asymmetric inlets are improved. The maximum increase is A_vm1 (TBSS)_ = 7.82 (1/s) in the CS structure, while the minimum increase is A_vm1 (CSS)_ = 4.89 (1/s) in the CSS structure. The increase in initial vorticity proves that the asymmetrically placed inlets optimization strategy is feasible. However, the difference in the range of increasing shows that this optimization strategy needs to be used in conjunction with appropriate sidewall spiral patterns to exert the best effect. In section 2, the curve change trends can be summarized into two situations: amplification or decline. Similar to the SSH structure, the vorticity magnitude of CS (A_vm1 (CS)_ = 17.91 (1/s)) is further amplified and reaches the peak value of all 4 spiral patterns. However, the vorticity magnitude of CSS and TBSS are in significant decline: in CSS A_vm1 (CSS)_ = 4.89 (1/s) drops to A_vm2 (CSS)_ = 2.85 (1/s), and in TBSS A_vm1 (TBBS)_ = 5.87 (1/s) drops to A_vm2 (TBBS)_ = 2.85 (1/s). After passing through section 2, all the vorticity magnitude curves of sidewall spiral patterns in Figure 5 show a downward trend, but the rates downward are different. The CS curve tends to decrease almost linearly, but due to the peak value established in section 2, the vorticity magnitude is still maintaining an advantage at the mixer outlet (A_vm5 (CS)_ = 4 (1/s)). While the other 2 vorticity magnitude curves show a gentle downward trend. The outlet vortex strengths of TBSS (A_vm5 (TBSS)_ = 1.01 (1/s)) are similar to that of SSH (A_vm5 (SSH)_ = 1.39 (1/s)) and obviously higher than that of CSS (A_vm5 (CSS)_ = 0.30 (1/s)).

**Figure 7:**
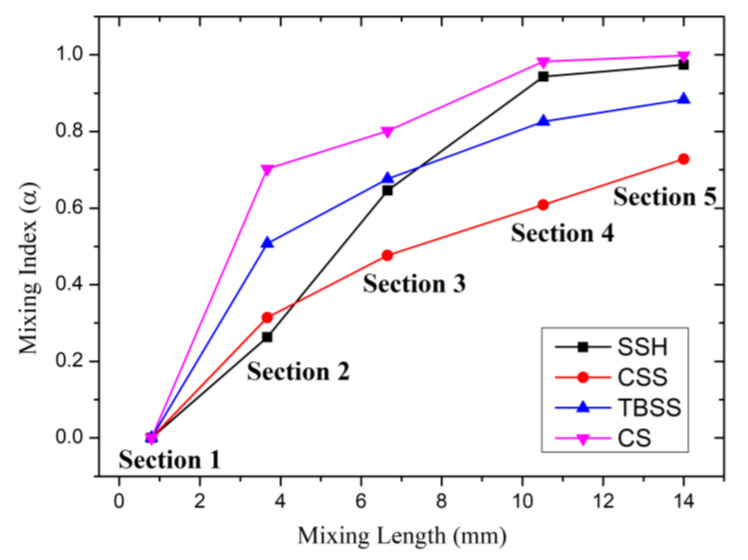
The mixing index curves of micromixer with different spiral patterns.

To reveal the relationship between vortex magnitude and mixing effect, according to Equation (4), the mixing index curves of different sidewall spiral patterns when *u* = 5 × 10^−3^ (m/s) are shown in Figure 7. The positions of the sampling sections in the curve are still the same as in Figure 2. section 1 is close to the inlet of the mixing chamber, and the promotion time of vortexes for liquid mixing is too short, so section 1 is treated as the unmixed reference, and the mixing indexes of all curves are 0. In section 2, after being promoted by the initial vortexes and three groups of wall spiral structures, the mixing index increases rapidly. The mixing index of CS is the highest (*α*_2 (CS)_ = 70.26%), SSH is the lowest (*α*_2 (SSH)_ = 26.33%), and TBSS (*α*_2 (TBSS)_ = 50.78%) and CSS (*α*_2 (CSS)_ = 31.4%) ranked second and third, respectively, which is consistent with the order of vortex magnitude of section 1 in Figure 6. Therefore, there seems to be a direct effect of vortex magnitude on the mixing index. In section 3, the consistent correspondence between the mixing index and vortex magnitude is broken. The mixing index of CS patterns still occupies the first place, which is consistent with the ranking of vortex magnitude. However, the mixing index of CSS (*α*_3 (CSS)_ = 47.68%) is the lowest of all 4 structures, but the vortex magnitude of CSS (A_vm3 (CSS)_ = 2.43 (1/s)) is slightly higher than that of SSH (A_vm3 (SSH)_ = 2.09(1/s)) and TBSS (A_vm3 (TBSS)_ = 2.06 (1/s)) in the same position. In section 4, the corresponding order between the mixing index and vortex magnitude changed again. The mixing index of SSH (*α*_4 (SSH)_ = 94.34%) rises to second place after CS (*α*_4 (CS)_ = 98.29%), which is consistent with the ranking of the vortex magnitude. The mixing index of CSS (*α*_4 (CSS)_ = 60.87%) remains the worst, but its vortex magnitude is still better than TBSS. In section 5, the mixing index ranking and the vortex magnitude correspond one-to-one again, and the order from big to small is CS (*α*_5 (CS)_ = 99.81%), SSH (*α*_5 (SSH)_ = 97.42%), TBSS (*α*_5 (TBSS)_ = 88.32%) and CSS (*α*_5 (CSS)_ = 72.79%). It can be seen from the above cross-section data analysis that there is a certain correlation between the mixing index and the vortex magnitude, but the vortex magnitude cannot completely determine the mixing results.

**Figure 8:**
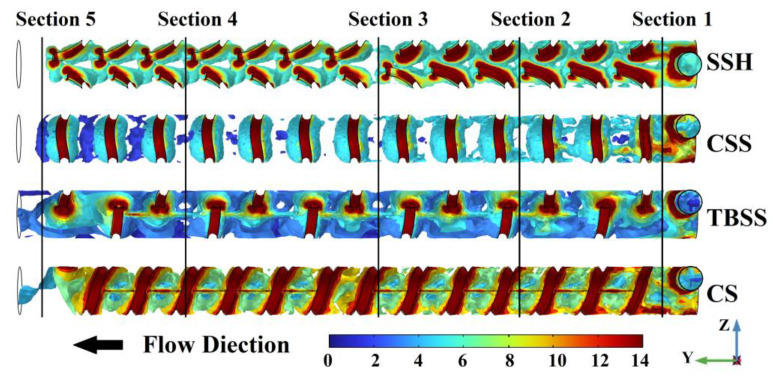
The 3D vorticity magnitude isosurface of micromixer with different spiral patterns.

In order to accurately reflect the vortex situation in the micromixer with different spiral patterns and further clarify the relationship between vortex magnitude and mixing index, the 3D vorticity magnitude isosurfaces are shown in Figure 8. The 3D vorticity magnitude isosurfaces are indicated by a rainbow color table, with dark red indicating the highest vorticity magnitude (14 (1/s)), while dark blue indicates the lowest (0 (1/s)). From the 3D vorticity magnitude isosurfaces of the SSH, the isosurfaces near the SSH structure on the wall are depicted in dark red, which indicates that the source of the vortices is located here. The vorticity magnitude isosurfaces in the mixing chamber are basically continuous along the *Y*-axis, except for the interruption caused by the structure reversing (near section 3). The isosurfaces are yellow-green before the structure reverses, while the isosurfaces are blue-green after the structure reverses. In addition, the lacunas between vorticity magnitude isosurfaces also increase obviously after the structure reversing. From the 3D vorticity magnitude isosurfaces of CSS, the CSS structure is still the source of the vortexes. However, the vorticity magnitude isosurfaces are confined around the CSS structure, which is no longer continuous in the Y direction of the mixing chamber. The discontinuity of the vortexes leads to a significant weakening of the mixing promotion. This is also the fundamental reason why the vortex strength of the CSS structure is comparable to other structures except for CS, while the mixing index is the worst. From the 3D vorticity magnitude isosurfaces of TBSS, the vorticity magnitude isosurfaces in the mixing chamber are also basically continuous along the *Y*-axis. But the dark red TBSS vortex sources are mainly connected by the blue isosurfaces, which indicates that the vorticity magnitude fluctuates greatly along the *Y*-axis and cannot maintain at a high level all the time. The gradient distribution of vorticity magnitude along the Z-axis that is caused by the staggered arrangement of TBSS is the fundamental reason for weakening the persistence along the *Y*-axis. From the 3D vorticity magnitude isosurfaces of CS, the vorticity magnitude is not only continuous along the *Y*-axis but also maintained at a high level all the time. So, the mixing index of CS is the best among all structures. From the above analysis, it is clear that not only higher strength is required to obtain a better mixing index, but the continuity of vorticity magnitude along the direction of liquid moving is also an important factor.

**Figure 9:**
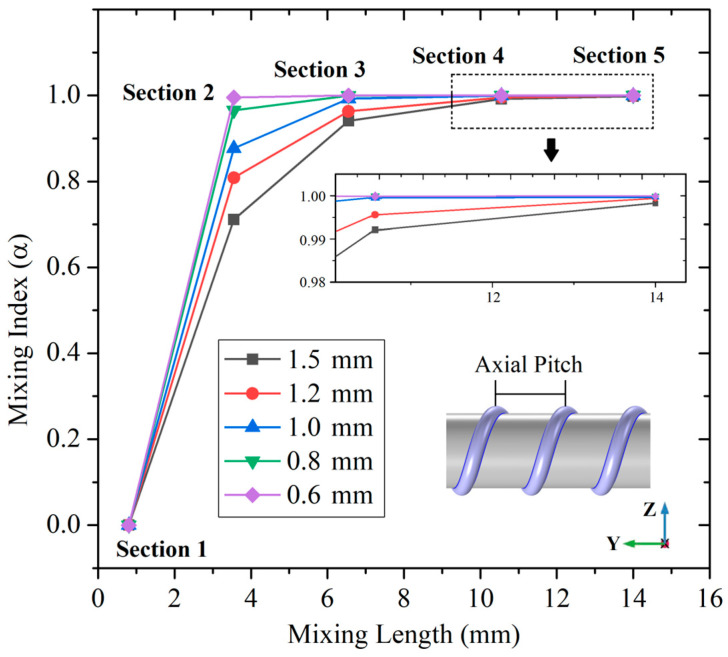
The mixing index curves of CS micromixer with different spiral axial pitches.

Then, a series of numerical simulations about the CS mixer is carried out to analyze the influence of spiral pattern structure parameters on the mixing index. When *u* = 5 × 10^−3^ m/s, the mixing index curves of the CS micromixer with different spiral axial pitch (AP) are shown in Figure 9. The positions of the sampling sections in the curves are still the same as in Figure 2. The changing of the spiral axial pitch means that the turns of CS structures will change under a certain mixing length (14 mm). The relationships between the spiral axial pitch and the number of turns are shown in Table 2.

Combined with Figure 9 and Table 2, when AP = 1.5 mm, the mixing index reaches 99.2% after 6 turns at section 4, which is close to uniform mixing. When AP = 1.2 mm, the mixing index reaches 99.56% after 7.5 turns at section 4, which is also close to uniform mixing. When AP = 1 mm, the mixing index reaches 99.27% after 6 turns at section 3, which shows that uniform mixing can be realized in a shorter mixing length. When AP = 0.8 mm, the mixing index reaches 99.94% after 7.5 turns in section 3, the mixing length to achieve uniform mixing is similar to AP = 1 mm. When AP = 0.6 mm, the mixing index reaches 99.52% after 5 turns at section 2, and the mixing length to achieve uniform mixing is further shortened. The above changes show that, when the mixing length is consistent, the denser turns of the sidewall spiral, the better mixing. However, no matter what the turn density, the CS mixer can achieve uniform mixing within the mixer length of 14 mm.

**Figure 10:**
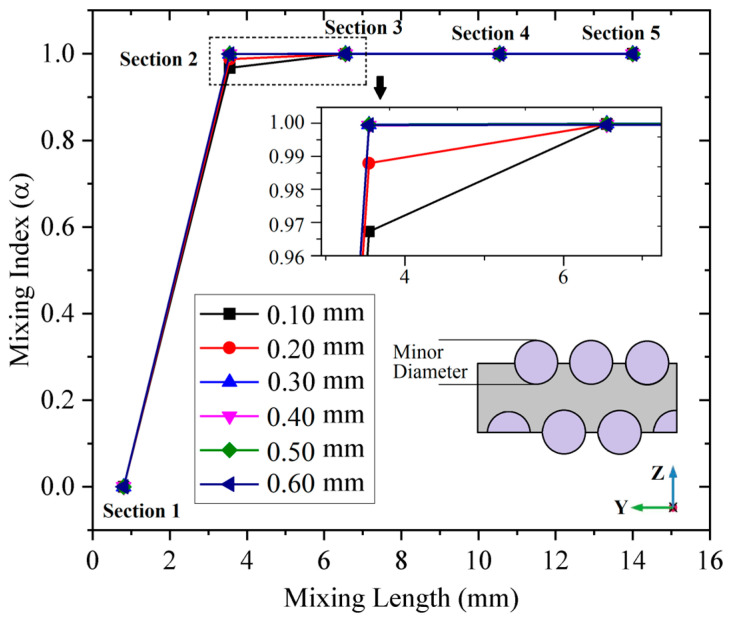
The mixing index curves of CS micromixer with different spiral minor diameters.

Then, the relationship between the minor diameter of the sidewall spiral and the mixing index is discussed. The positions of sampling sections in the curves are also the same as in Figure 2. As shown in Figure 10 in section 2, when the minor diameter (MD) is 0.1 mm, the mixing index is 96.74%, when MD = 0.2 mm, the mixing index is 98.81%, and when MD ≥ 0.3 mm, the mixing index is more than 99%, the uniform mixing is realized. So, when it is less than 0.3 mm, there is a promotion of uniform mixing that is caused by sidewall spiral MD increasing. But in section 3, all the mixing indexes of CS mixers with different MD are more than 99% and achieve a uniform mixing, which shows that there is only a little promotion of sidewall spiral MD on the mixing index.

**Figure 11:**
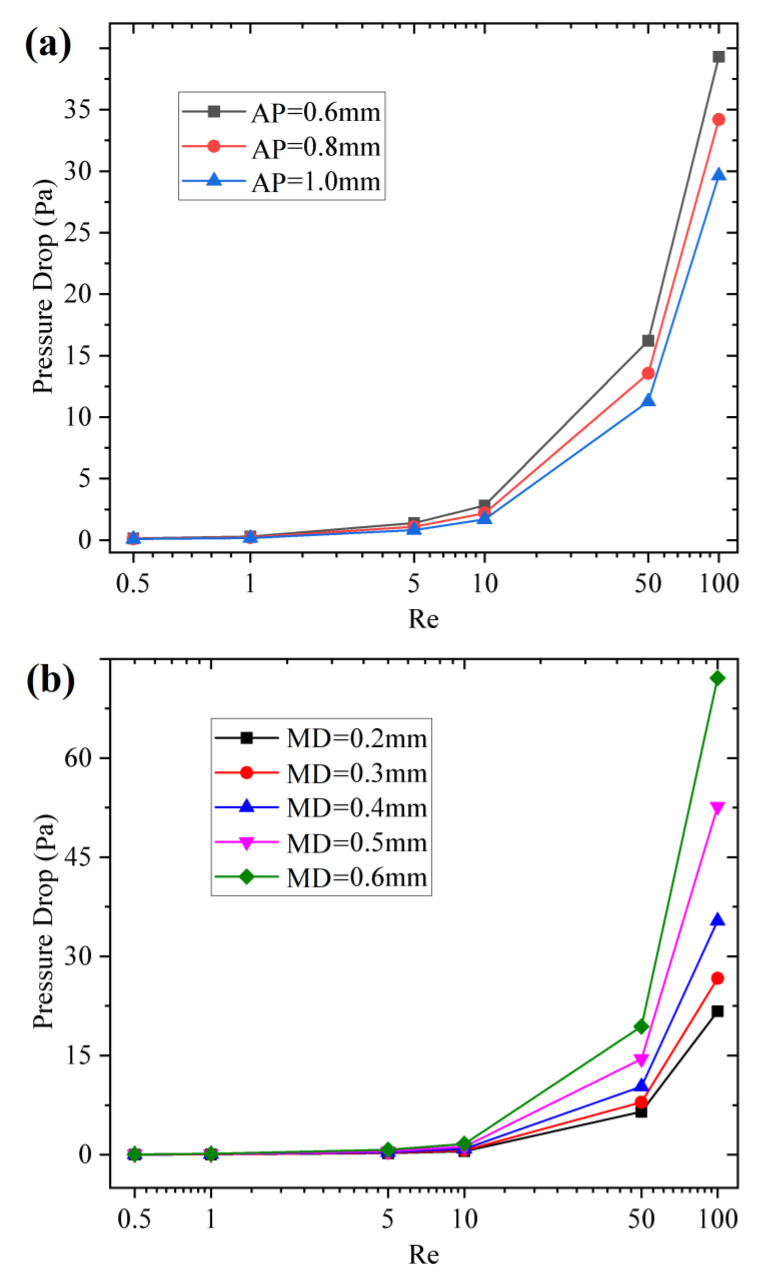
The pressure drop curves of CS micromixer with different spiral pattern structure parameters. (**a**) Different spiral axial pitch; (**b**) different spiral minor diameters.

In addition to the mixing index, the energy loss, also known as driving pressure drop, is another important indicator of passive micromixers to be considered. The pressure drop experienced in a micromixer is intrinsically tied to the amount of the difference between the area-average total pressures at the inlet and outlet of the micromixer and can be calculated directly by Comsol Multiphysics 6.0. By increasing the flow rate(*u*) from 5 × 10^−4^ m/s to 1 × 10^−1^ m/s, correspondingly, the Reynolds number (Re) increased from 0.5 to 100. Under different Re, the influence of sidewall spiral pattern structure parameters on driving pressure drop is investigated and shown in Figure 11.

In Figure 11a, if the Re and MD are constant, the pressure drop (PD) is inversely proportional to AP. For example Re = 5 and MD = 0.2 mm, the PD decreases from 0.334 Pa to 0.235 Pa when the AP increases from 0.6 mm to 1.0 mm. The AP increase leads to a decrease in sidewall spiral turns. When moving in a mixing chamber with relatively smooth side walls, the energy loss caused by wall friction will be reduced and so is the PD loss. If the AP and MD are constant, the PD is proportional to Re. For example AP = 1 mm and MD = 0.2 mm, the PD increases from 0.023 Pa to 21.69 Pa when the Re increases from 0.5 to 100. The Re increase represents the greater initial energy that must be provided at the mixer inlet. So, the PD also increases when passing through the same sidewall spiral patterns [38]. In Figure 11b, if the Re and AP are constant, the PD is proportional to MD. For example Re = 5 and AP = 1 mm, the PD increases from 0.235 Pa to 0.726 Pa when the MD increases from 0.2 mm to 0.6 mm. The MD increase leads to an increase in sidewall pattern roughness. When moving in a mixing chamber with a coarser side wall, the energy loss caused by wall friction will be increased and so the PD increase. Obviously, the sidewall spiral structural parameters will both affect the mixing index and pressure drop. When AP decreases, the denser turns of the sidewall spiral and the mixing index improved, but the pressure drop of the mixer also increased. When MD increases, the sidewall spiral is rougher, but the improvement of the mixing index is limited, while the pressure drop increases obviously. The pressure drop should be reduced as much as possible on the basis of ensuring the mixing index, so the AP = 1 mm and MD = 0.2 mm are selected as the final sidewall spiral structural parameters. When Re = 5, the mixing index of the CS mixer with the above structural parameters can reach 99.27% after a 7 mm mixing distance, and the PD is only 0.187 Pa.

### 3.2. Mixer Chip Manufacture and Performance Test

**Figure 12:**
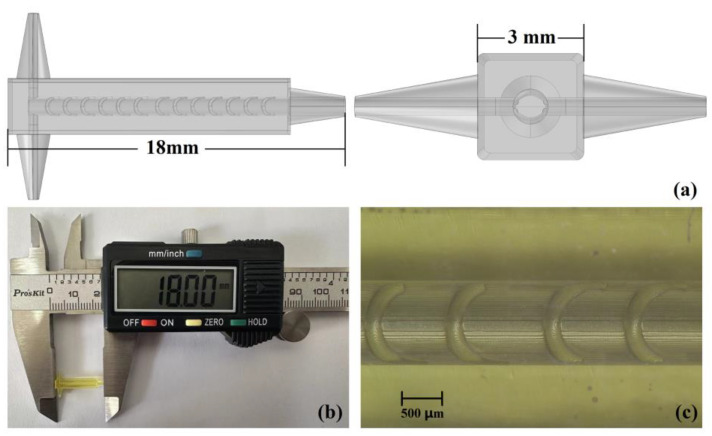
Structure diagram of SSH mixer chip and prepared chip photo. (**a**) SSH micromixer chip structural design drawing; (**b**) full-view photograph of SSH mixer chip; (**c**) detailed photograph of SSH structure.

In order to characterize the mixing situation in the cylindrical mixing chamber more accurately by the visual test method, the chip is designed as a square column, as shown in Figure 12a. The structural detail of SSH is shown in Figure 1, as well as TBSS, CSS and CS as shown in Figure 5, the micromixer chips are fabricated by nanoArch^®^ P150 printing system (BMF Precision Tech Inc., Shenzhen, China) [39]. The chip preparation processes are as follows:

1. A series of 2D bitmap files with special patterns were obtained by slicing 3D micromixer structural design drawings;
2. Based on the above 2D bitmap files, the digital dynamic masks are generated by the digital micro-mirror device (DMD) in the nanoArch^®^ P150 printing system;
3. When a specific wavelength of ultraviolet light (UV at 405 nm) passed through the digital dynamic masks, the photosensitive resin materials are exposed and cured. One precision structure layer can be produced by one exposure;
4. The micromixer chip is obtained by accumulating the structure after layering solidification. The photographs of the micromixer chip are shown in Figure 12b,c.

Before the test, the flow velocity *u* = 5 × 10^−3^ m/s must convert to volumetric flow *Q* (m^3^/s) according to Equation (6). Here, *L* is the characteristic length of the cylindrical mixing chamber, *η*_0_ is the viscosity of the dyed water (8 × 10^−4^ Pa s at 25 °C) and *ρ* is the density of water (998 kg/m^3^). When *u* = 5 × 10^−3^ m/s, in the micromixer inlet, the structural characteristic scale is 5 × 10^−4^ m, so Re = 5 and *Q* = 1.2 × 10^−1^ mL/min.

(6) Q=ReLη0ρ

**Figure 13:**
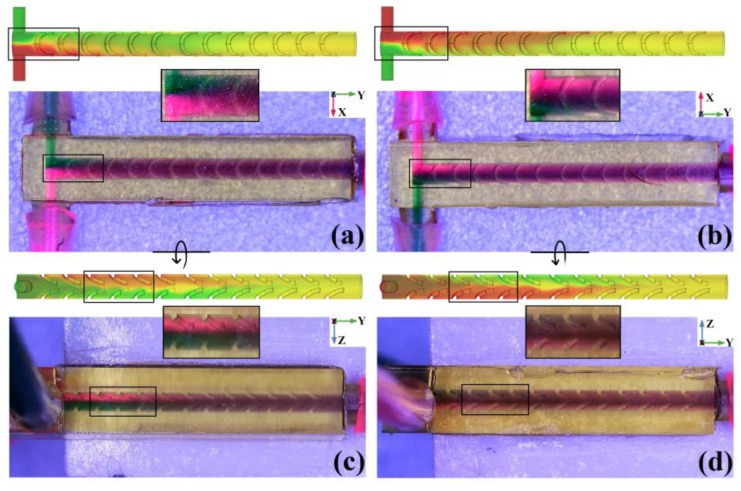
Simulation concentration distribution and microscopic visual test of SSH micromixer. (**a**) Top view; (**b**) bottom view; (**c**) left view; (**d**) right view.

Then, 1 mol/L Rhodamine B aqueous solution (red) and 1 mol/L methyl green 1 mol/L rhodamine B aqueous solution (green) are utilized as indicators and the viscosity of indicators solution can be approximated as water 1 × 10^3^ N·s/m^2^; the dual channel micro-injection pump (LSP02-1B, Longer Precision Pump Co., Ltd., Baoding, China) provides impetus and controlled volumetric flow; the microscope (GP-490H, Kunshan Gaopin Precision Instrument Co., Ltd., Kunshan, China) is used to observe the mixing progress of micromixer with different sidewall special patterns. When *Q* = 1.2 × 10^−1^ mL/min (Re = 5), the comparison of simulated concentration distribution and microscopic visual test of micromixers with SSH sidewall patterns are shown in Figure 13. Because the chip material is a photosensitive resin material with high transparency, the microscope backlight will penetrate the chip and be captured by the CCD sensor. So, the visual test result is the superposition of the indicator’s color in the *Z*-axis direction or *X*-axis direction. In order to compare the simulation and test results more accurately, the surface concentration distribution is set to transparent form in COMSOL Multiphysics 6.0 software.

In the top view of SSH as shown in Figure 13a, when the green and red liquids just enter the mixing chamber, the concentration distribution can be regarded as an extension of the symmetrical T-shaped inlet. The interface between the two colors is clearly visible and located in the Y-Z plane of the mixing chamber’s central axis (marked with a black rectangle). After undergoing 2 groups of SSH structures, the green liquid quickly covers the surface of the red liquid, so that the color of the mixing chamber observed from the top view changes to deep purple. As the passing SSH structures increase, the top view liquid color tends to be consistent purple, which shows that the mixing effect is well. In the bottom view of SSH, as shown in Figure 13b, the observed mixing process is just opposite to the top view. Near the mixer inlet, the interface of two colors is still distinct but the positions are interchanged (marked with a black rectangle). After undergoing 2 groups of SSH structures, the red liquid quickly covers the surface of the green liquid. And then, with the passing SSH structures increase, the bottom view liquid color tends to be consistent purple also. The left view of the SSH micromixer, as shown in Figure 13c, can be obtained by flipping the top view according to the arrow direction. After 2 groups of SSH structures, a clear interface between red liquid and green liquid in the mixing chamber Z direction can be observed from the left view (marked with a black rectangle). This phenomenon is caused by the liquid rotation under the action of the SSH sidewall structure. With the passing SSH structures increase, the liquid interface in the left view also gradually disappears and eventually turns purple. The right view of the SSH micromixer, as shown in Figure 13d, can be obtained by flipping the bottom view according to the arrow direction. After 2 groups of SSH structures, a clear interface between red liquid and green liquid in the mixing chamber Z direction can be observed from the right view also (marked with a black rectangle). When the passing SSH structures increase, the liquid interface in the right view also gradually disappears and eventually turns purple. From the comparative analysis of simulation results and visual tests in Figure 13, the simulation results are in good agreement with the visual test.

**Figure 14:**
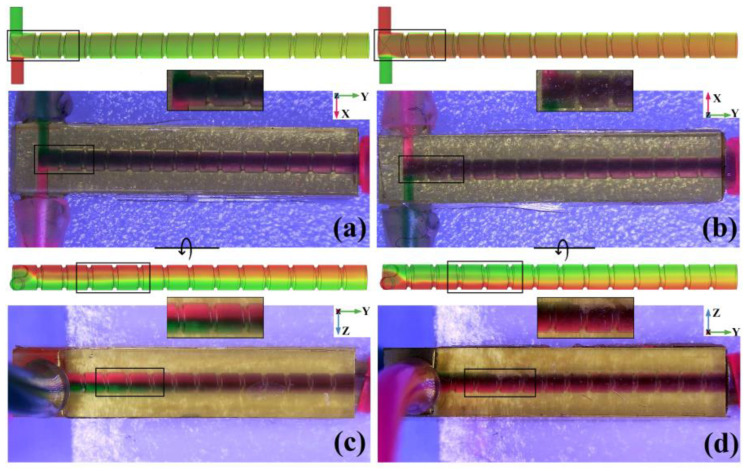
Simulation concentration distribution and microscopic visual test of CSS micromixer. (**a**) Top view; (**b**) bottom view; (**c**) left view; (**d**) right view.

According to the visual test flow of the SSH micromixer, the CSS TBSS and CS structure micromixers are tested in turn. From the top view of the CSS mixer as shown in Figure 14a, when entering the mixing chamber, the green liquid quickly covers the red liquid, because the green liquid inlet is placed at a higher position in the Z direction of the mixing chamber (marked with a black rectangle). In Figure 14b, the bottom view of the CSS mixer, the red liquid quickly covers the green liquid, which is also due to the asymmetric inlet. With the passing CSS structures increase, both the top view and the bottom view of the CSS mixer gradually changed from deep purple to purple, but the interface between the two different purple persisted to the mixer outlet. In the left and right view of the CSS mixer, as shown in Figure 14c,d, near the chamber inlet, the interface of red and green liquid in the *Z*-axis direction is obvious (marked with a black rectangle). It indicates that the asymmetric inlet makes the liquid rotate immediately when entering the mixing chamber. With the passing CSS structures increase, the overall color of the mixing chamber approaches purple, but is not uniform up to the chamber outlet. The above visual test results show that asymmetric inlets can provide larger initial vortexes, but CSS structures have limited amplification on them.

**Figure 15:**
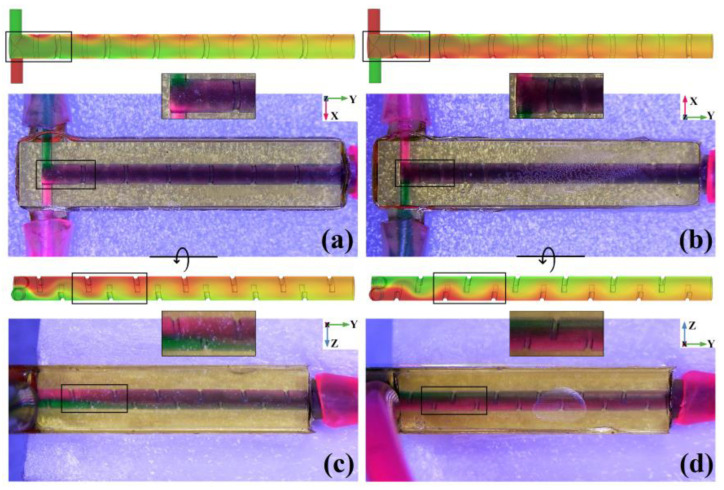
Simulation concentration distribution and microscopic visual test of TBSS micromixer. (**a**) Top view; (**b**) bottom view; (**c**) left view; (**d**) right view.

From the top view and bottom view of the TBSS mixer as shown in Figure 15a,b, if the liquid enters the chamber through an inlet with a higher position in the Z direction, it will quickly cover liquid that enters the chamber through the lower inlet (marked with a black rectangle). Subsequently, no matter from the top or bottom view, the liquid in the mixing chamber appears deep purple, and the mixer seems to achieve an ideal result. However, from the left view and right view of the TBSS mixer as shown in Figure 15c,d, the layering of the green and red liquid in the Z direction near the inlets is obvious (marked with a black rectangle), and still can be distinguished until the outlet. The side view’s experimental results show that the mixing results of the TBSS mixer are not as ideal as that observed from the top view or bottom view.

**Figure 16:**
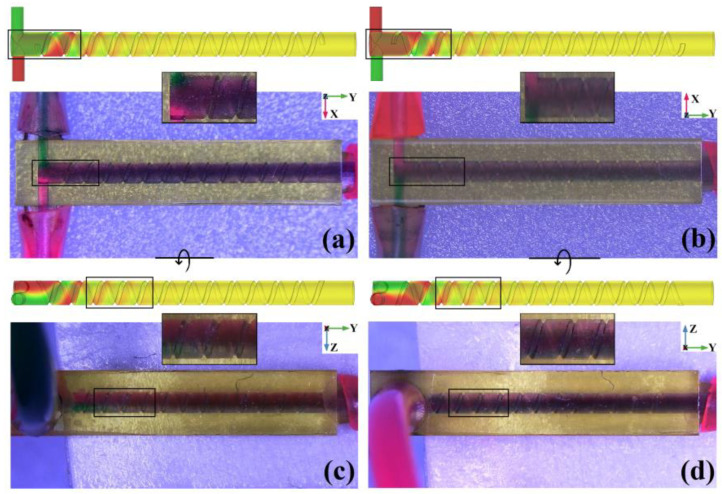
Simulation concentration distribution and microscopic visual test of CS micromixer. (**a**) Top view; (**b**) bottom view; (**c**) left view; (**d**) right view.

From the top view and bottom view of the CS mixer as shown in Figure 16a,b, except for a distinguishable interface between red and green liquids near the inlets (marked with a black rectangle), the other positions of the mixing chamber are purple. In the left and right view of the CSS mixer as shown in Figure 16c,d, except for a fuzzy interface between red and green liquids near the inlets (marked with a black rectangle), the other positions of the mixing chamber are also uniform purple. The visual test results in the 4 directions of the mixing chamber all prove that the performance of the CS mixer is very good.

## 4. Conclusions

From the digital simulation research on SSH, CSS, TBSS and CS mixers, the vortex magnitude used to characterize the initial vortexes magnification effect is mainly determined by the shear rate in the mixing cavity. The generation of shear rate is attributed to the velocity gradient in the cross-section perpendicular to the flow direction (Y direction), and this velocity gradient is related to the spiral pattern structural distribution variation on the sidewall. Therefore, the initial vortex can be amplified by optimizing the spiral pattern on the mixing chamber sidewall, and the mixing performance can also be improved. After in-depth analysis, it is found that the relationship between vortex magnitude and mixing index of mixers with different spiral patterns is not a simple direct proportion. There is another important determinant factor for the mixing index: the continuity of vorticity magnitude along the liquid flow direction. If the side wall spiral structure continuity of the mixing chamber is poor, even if this structure can induce a large vorticity magnitude, the final mixing result will be affected too. For example, with a CSS mixer, the initial vortex magnitude near the inlet (A_vm1 (CSS)_) is 4.89 (1/s), but the mixing index near the outlet (*α*5 _(CSS)_) is only 72.79% when Re = 5. If the vorticity magnitude has good continuity, even if the initial vortex magnitude is not well, the mixing index will be good, too. For instance, with a SSH mixer, the initial vortex magnitude near the inlet (A_vm1 (SSH)_) is only 4.02 (1/s), while the mixing index near the outlet (*α*5 _(SSH)_) reached 97.42% when Re = 5. If a structure can induce a large initial vorticity magnitude and have excellent continuity, such as a CS mixer, the mixing result is definitely the best (*α*5 _(CS)_ = 99.81%). In addition, the specific structure parameters such as spiral axial pitch and minor diameters have little effect on the mixing index and pressure drop. From the visual tests on SSH, CSS, TBSS and CS mixers, the results show good agreement with the simulation. Moreover, the square column mixer chip appearance design is helpful to comprehensively reflect the effect of the mixer and avoid the test effect deviation caused by a single perspective.

## Figures and Tables

**Table 1:** The basic parameters of the simulation model.

Parameters Name	Value
Fluid Density (*ρ*)	1 × 10^3^ (kg/m^3^)
Dynamic Viscosity Coefficient (*η*)	1 × 10^−3^ (Pa·s)
Flow Velocity (*u*)	5 × 10^−3^ (m/s)
Viscosity (*μ*)	1 × 10^3^ (N·s/m^2^)
Diffusion Coefficient (*D*)	1 × 10^−9^ (m^2^/s)

**Table 2:** The relationships between spiral axial pitch and turns.

Spiral Axial Pitch (mm)	Number of CS Structure Turns
1.5	8
1.2	10
1	12
0.8	15
0.6	20
